# Xylanolytic Extremozymes Retrieved From Environmental Metagenomes: Characteristics, Genetic Engineering, and Applications

**DOI:** 10.3389/fmicb.2020.551109

**Published:** 2020-09-17

**Authors:** Digvijay Verma, Tulasi Satyanarayana

**Affiliations:** ^1^Department of Microbiology, Babasaheb Bhimrao Ambedkar (Central) University, Lucknow, India; ^2^Department of Biological Sciences and Engineering, Netaji Subhas University of Technology, Dwarka, New Delhi, India

**Keywords:** extremozymes, xylanolytic enzymes, metagenomes, metagenomics, GH-10 and GH-11 xylanases, genetic engineering

## Abstract

Xylanolytic enzymes have extensive applications in paper, food, and feed, pharmaceutical, and biofuel industries. These industries demand xylanases that are functional under extreme conditions, such as high temperature, acidic/alkaline pH, and others, which are prevailing in bioprocessing industries. Despite the availability of several xylan-hydrolyzing enzymes from cultured microbes, there is a huge gap between what is available and what industries require. DNA manipulations as well as protein-engineering techniques are also not quite satisfactory in generating xylan-hydrolyzing extremozymes. With a compound annual growth rate of 6.6% of xylan-hydrolyzing enzymes in the global market, there is a need for xylanolytic extremozymes. Therefore, metagenomic approaches have been employed to uncover hidden xylanolytic genes that were earlier inaccessible in culture-dependent approaches. Appreciable success has been achieved in retrieving several unusual xylanolytic enzymes with novel and desirable characteristics from different extreme environments using functional and sequence-based metagenomic approaches. Moreover, the Carbohydrate Active Enzymes database includes approximately 400 GH-10 and GH-11 unclassified xylanases. This review discusses sources, characteristics, and applications of xylanolytic enzymes obtained through metagenomic approaches and their amelioration by genetic engineering techniques.

## Introduction

**FIGURE 1:**
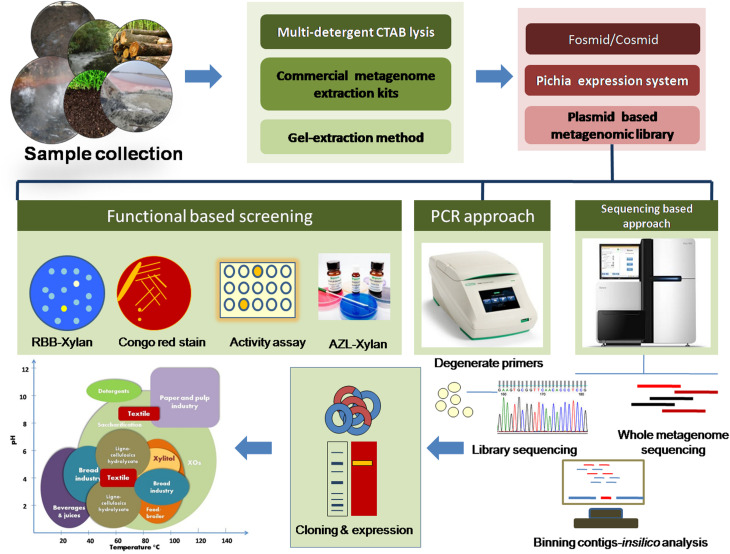
Schematic representation for retrieving xylan-degrading enzymes using metagenomic approaches.

Extensive biotechnological applications of xylanolytic extremozymes have raised interest as well as their demand in several industrial processes. Extremozymes are expected to withstand extreme conditions posed during the downstream processing of lignocellulosic materials. For example, thermo-alkali-stable xylanases are well suited for the paper industry for their use in bio-bleaching of paper pulp (Kumar et al., 2016; Figure 1). The bread industry prefers xylanases/β-xylosidases of thermo-acid stable properties (Uhl and Daniel, 1999; Harris and Ramalingam, 2010; Kumar et al., 2016). Salt-tolerant hemicellulases are the choice of seafood processing industries (Al-Darkazali et al., 2017). Similarly, barophilic, psychrophilic, and xylan-degrading enzymes functional at low water activity find multiple applications in food and beverage processing. Microorganisms exhibit a high degree of genomic and metabolic flexibility for adapting to extreme environmental conditions, and therefore, they occur ubiquitously and act as a reservoir for proteins/enzymes capable of withstanding extreme environmental conditions (Badhai et al., 2015). Several extreme environments have been explored for obtaining extremophilic microbes for their exploitation in harnessing extremozymes (Shi H. et al., 2013; Wang et al., 2015). Many xylan-depolymerizing enzymes have also been identified from innumerable prokaryotic and eukaryotic microorganisms from extreme environments (Kumar et al., 2013a; Verma et al., 2019). However, the limitations associated with these microbial enzymes cannot be ignored. Xylanolytic enzymes of bacterial origin are mostly preferred over the fungal xylanases due to the retention of activity by the former under extreme environmental conditions (Kumar et al., 2016). Broad pH and temperature optima and fair stability under extreme conditions in addition to broad substrate specificity make the bacterial xylanases preferable over those from fungi although the production levels are of the former are lower than those of the latter (Verma et al., 2019).

Due to the problems encountered in simulating environmental conditions in the laboratory and limited culturability, we lose a major chunk of microorganisms present in environmental samples. Metagenomics has emerged as an alternative tool for discovering novel bioactive molecules from various environments (Lorenz and Eck, 2005; Berini et al., 2017; Fredriksen et al., 2019). Several novel xylanolytic enzymes with unique properties have also been obtained from various extreme environmental samples using metagenomic approaches. Wang et al. (2016) further emphasize digging out the entire enzyme assemblages from environments that can depolymerize the target complex polysaccharides efficiently. Such a cocktail of enzymes usually share similar biophysical properties due to their common origin, which further enhances their compatibility for complete substrate hydrolysis (Berini et al., 2017). Several such lignocellulases have been successfully derived using the metagenomic approaches (Thidarat et al., 2012; Wang et al., 2015; Liu et al., 2018). Unfortunately, a majority of the discovered xylanolytic enzymes are still uncharacterized, which can be seen in the Carbohydrate Active Enzymes (CAZy) database^1^. In addition, most of those characterized are limited to their biophysical properties only and not well explored for their applicability under industrial process conditions. This review summarizes various facets of metagenomic xylanolytic extremozymes, such as their characteristics, comparison with the available xylan-degrading enzymes, improvement by genetic/protein engineering, and potential applications.

## Timeline of Metagenomic Xylanolytic Enzyme Discovery

It took nearly 10 years to report the first xylan-degrading enzyme through the metagenomic approach after its introduction. Sunna and Bergquist (2003) were the pioneers who reported the first xylanase from the hot pool metagenome using a genome-walking PCR (GW-PCR) approach. The retrieved xylanase shared similarity with GH-10 xylanase with unusual features; thus, this can be considered the first report of the GH-10 family xylanase of metagenomic origin. It was followed by Brennan et al. (2004), who reported the first GH-11 and GH-8 xylanases from the insect gut metagenome using direct cloning of the environmental DNA. Lee et al. (2006) reported the second GH-8 xylanase from the lagoon of a dairy farm. The first multifunctional glycosyl hydrolases of GH-5 and GH-26 were reported by Palackal et al. (2007). Attempts have been made for the first time to enhance the sensitivity of PCR using rolling circle amplification of the horse gut metagenome that revealed five GH-10 xylanases (Yamada et al., 2008). Of various environmental samples, the soil was first explored for cloning community DNA by Hu et al. (2008) to uncover hidden metagenomic xylanases. Similarly, Kwon et al. (2010) investigated the compost soil metagenome and reported five xylanases out of 12,380 fosmid clones. The claim of Jeong et al. (2012) for reporting the first xylanase from the compost metagenomic library, therefore, stands corrected. Verma et al. (2013a) first reported an industrially relevant metagenomic endoxylanase that finds applicability in bio-bleaching of pulp samples.

A novel xylanosome operon having three contiguous genes of family GH-8, GH-10, and GH-11 was identified from a wood-feeding termite gut metagenome in 2012 (Thidarat et al., 2012). Further, Zhang et al. (2014) studied the role of human gut inhabitant Bacteroidetes by reporting two GH-10 family endoxylanases from the human gut metagenome. Several new findings associated with the xylan-depolymerizing enzymes of metagenomic origin have been published during the past 5 years (Wang et al., 2015; Rashamuse et al., 2016; Kim et al., 2018; Ellila et al., 2019; Alves et al., 2020; Victorica et al., 2020; Tables 1, 2). Recently, a novel computational method, thermal activity prediction for xylanase (TAXyl), was developed to predict the thermal activity of GH-10 and GH-11 xylanases (Shahraki et al., 2019). Although several xylan-degrading enzymes are known today, using culture-independent metagenomic approaches, less than 100 of them have been adequately characterized. Therefore, there is an urgent need to characterize the remaining metagenomic xylanases.

**TABLE 1:** An update of metagenomic GH-10 and GH-11 endoxylanases.

S. No.	Source	Name	Vector/host/approaches	Library type/approach	Positive hits	Opt_*pH*_	Opt_*Temp*_.(°C)	Molecular mass (kDa)	Family	References
**1.**	Camel rumen metagenome	PersiXyn1	NA	Direct sequencing		8.0	40	43	GH-11	Ariaeenejad et al., 2019
**2.**	Compost metagenome	XYL21, XYL38	CopyControl Fosmid Library Production kit	Fosmid	40	5.5–7.0	80 (XYL38)	41.9 (XYL38)	GH-10	Ellila et al., 2019
**3.**	Arctic mid-ocean ridge vent	AMOR_GH10A	Metagenomic data set	NA	NA	5.6	80		GH-10	Fredriksen et al., 2019
**4.**	Camel rumen metagenome	XylCMS	454 Pyrosequencing	NA	NA	6.0	55	46	GH-11	Ghadikolaei et al., 2019
**5.**	Lobios hot spring sediment	XynA3	Fosmid	Fosmid	1/150,000	6.5	80	41	GH-11	Knapik et al., 2019
**6.**	Saline-alkaline soil	Xyn22	pMD18T/*E*. *coli* DH5α	Plasmid	150/–	7.0	60	25	GH-11	Li et al., 2019
**7.**	Termite gut metagenome		CopyControl fosmid library production kit	Fosmid	101/50,000	ORF7: 7.0 ORF21:7.0	ORF7: 60 ORF21: 60	ORF7: 45 ORF21: 53.1	GH-10	Liu et al., 2019
**8.**	Soda lake Dabusu metagenome	-NA-	Illumina HiSeq2500	NA	NA	NA	NA	NA	NA	Wang et al., 2019
**9.**	Black goat rumen	KG42	CopyControl fosmid library production kit	Fosmid	17/115,200	5.0	50	41	GH-10	Kim et al., 2018
**10.**	Termite gut metagenome	Xyl1	CopyControl fosmid library production kit	Fosmid	14/40,000	6.0	50	55	GH-11	Rashamuse et al., 2016
**11.**	Hu sheep rumen metagenome	xyn-lxy	EpiFOSTM Fosmid Library Production Kit	Fosmid	18/12704	6.0	50	80	GH-10	Wang et al., 2015
**12.**	Cattle rumen metagenome	Xyln-SH1	EpiFOSTM Fosmid Library Production Kit	Fosmid	1/–	6.5	40	41.5	GH-10	Cheng et al., 2012
**13.**	Compost soil metagenome	Mxyl	p18GFP vector	Plasmid	1/36,000	9.0	80	43	GH-11	Verma et al., 2013b
**14.**	Chicken cecum metagenome	XynAMG1	CopyControl fosmid library production kit	Fosmid	NM	6.0	45	40	GH-10	Al-Darkazali et al., 2017
**15.**	Cow dung compost	xyn10CD18	PCR approach using degenerate primers	NA	NA	7.0	75	43.3	GH-10	Sun et al., 2015
**16.**	Soil	XynH	pHBM803 and pHBM625/*E. coli* BL21(DE3) and XL10-Gold	Plasmid	1/24,000	7.8	40	39	GH-10	Hu et al., 2008
**17.**	Compost metagenome	Xyn10J	CopyControl fosmid library production kit	Fosmid	5/–	7.0	40	40	GH-10	
**18.**	Compost metagenome	X1, X2, X3, X4, and X5	CopyControl fosmid library production kit	Fosmid	5/12,380	5.5–6.0	50–55	ND	ND	Kwon et al., 2010
**19.**	Soil and pulp enrichment culture	X2P1	pUC19/*E. coli* DH5α	Plasmid	1/40,000	ND	ND	ND	ND	Mori et al., 2014
**20.**	*Globitermesbrachycerastes* gut metagenome	Xyn7	Fosmid library	Fosmid	ND	7	55		GH-11	Qian et al., 2015
**21.**	Termite gut metagenome	xynGH11-7	PCR approach using degenerate primers	NA	NA	6	30	26.7	GH-11 and many more	Sheng et al., 2015
**22.**	Alpine Tundra soil	NM	PCR approach using degenerate primers	NA	NA	ND	ND	ND	ND	Wang et al., 2010
**23.**	POME (Pal oil mill effluent)	-NM-	CopyControl fosmid library production kit	Fosmid	6/–	NM	NM	NM	GH-10 and others	Benbelgacem et al., 2018
**24.**	Fungus-growing termite, *Pseudacanthotermes militaris*	Xyn3	CopyControl fosmid library production kit	Fosmid	42/16,000-gut 12/24,000-comb	NM	NM	NM	NM	Bastien et al., 2013
**25.**	Goats ruminal liquid metagenome	Multiple xylanolytic enzymes	CopyControl fosmid library production kit	Fosmid	22/15,0000 endo-xylanase	5–7	50	ND	GH-11	Duque et al., 2018
**26.**	Thermophilic methanogenic digester community enriched with paper	Multiple xylanolytic enzymes	CopyControl fosmid library production kit	Fosmid	37/9700	5–6	60–75	ND	GH-11	Wang et al., 2015
**27.**	Kinema soyabean metagenome	NM	Whole metagenome sequencing	NA	NA	ND	ND	ND	GH-11	Kumar et al., 2019
**28.**	Grass hay-fed dairy cow rumen metagenome	xyn10N18	BAC library	BAC	ND	6.5	35	54.5	GH-10	Gong et al., 2013
**29.**	Wood-feeding higher termite	X1098.3	pCC1FOS	Fosmid	12/88,000	8	55	ND	GH-10 and 11	Thidarat et al., 2012
**30.**	Sugarcane bagasse	Xyn11 One of 5	CopyControl fosmid library production kit	Fomid	05/120,000	6	80	45	GH-11	Kanokratana et al., 2014
**31.**	Hot springs, Tattapani, Chhattisgarh	Illumina HiSeq1000	NA	MG-RAST and SEED databases	NA	NA	NA	NA	NA	Kaushal et al., 2018
**32.**	Rice straw metagenome	Umxyn10A	pWEB:TNC Cosmid	Cosmid	1/12,000	6.5	75	44	GH-10	Mo et al., 2010
**33.**	Cow dung compost metagenome	Xyn10CD18	PCR approach using degenerate primers	NA	NA	7	75	43.3	GH-10	Sun et al., 2015
**34.**	Termite gut metagenome	XYL6806 XYL6807 XYL6805 XYL6419	pET101/D-TOPO vector	*E. coli* BL21	NM	5–6	50	NM	XYL6806: GH8 Rest were of GH11	Brennan et al., 2004
**35.**	Cow manure metagenome	manf-x10	Lambda ZAP II vector and Gigapack III Packaging Extract	Bacteriophage	1/2 × 10^5^ plaque units	7	40	50.3	GH-10	Li et al., 2009
**36.**	Pawan hot spring metagenome	*xyn* PW8	PCR approach using GH-11 primers	NA	NA	7.0	50	23.3	GH-11	Helianti, 2007
**37.**	Vermiform appendixes of horses metagenome	*XynVA1*	Inverse PCR (IPCR)	NA	NA	ND	ND	42–63	GH-10	Yamada et al., 2008
**38.**	Hot pool metagenome	NM	Genomic walking PCR (GWPCR)	NA	NA	6	100	ND	GH-10	Sunna and Bergquist, 2003
**38.**	Hot pool metagenome	NM	Genomic walking PCR (GWPCR)	NA	NA	6	100	ND	GH-10	Sunna and Bergquist, 2003

*NM, not mentioned; ND, not determined; NA, not applicable.*

**TABLE 2:** An update of metagenomic xylanolytic enzymes other than GH-10 and GH-11 xylanases.

S. No.	Source	Name	Vector/host/approaches	Library type/approach	Positive hits	Opt_*pH*_	Opt_*Temp*_. (°C)	Molecular weight (kDa)	Family	References
**1.**	Yak rumen metagenome	Rubg3A and Rubg3B	pWEB Cosmid (Epicentre, United States)	Cosmid	2/4000	4.5–5.5	35–40	80	GH-3	Bao et al., 2012
**2.**	Yak rumen metagenome	RuBGX1	pWEB Cosmid (Epicentre, United States)	Cosmid	1/5000	6	50	80	GH-3	Zhou et al., 2011
**3.**	Yak rumen metagenome	RuXyn1 and RuXyn2	pWEB Cosmid (Epicentre, United States)	Cosmid	NM	5.5–7.0	40–55	42 and 50	GH-43	Zhou et al., 2012
**4.**	Cow rumen metagenome	RUM630-BX	Lambda ZAP II Vector and Gigapack III Packaging Ext., Agilent	Lambda phage	NM	7.0	25	ND	GH-43	Jordan et al., 2016
**5.**	Termite gut metagenome	Xyl1 and xyl8	CopyControl fosmid library production kit	Fosmid	12/8/12,000	5–6	40–50	53–90	GH43 and GH-3	Liu et al., 2018
**6.**	Wheat straw degrading microbial consortium	xylM1989	Microbial consortium	NA	NA	8	20	37.5	GH-43	Maruthamuthu et al., 2017
**7.**	Soil	MeXyl31	CopyControl fosmid library production kit	Fosmid	1/50,000	5.5	45	77	GH-31	Matsuzawa et al., 2016
**8.**	Compost	Biof1_09	CopyControl fosmid library production kit	Fosmid	1/–	4.5	50	12.8	GH-43	Sae-Lee and Boonmee, 2014
**9.**	Hot spring	AR19M-311-2, AR19M-311-11 and, AR19M-311-21	Direct sequencing	NA	NA	5	50–90		GH- 1, GH-3, GH-31, and GH-43	Sato et al., 2017
**10.**	Compost starter mixer	deAX	Lambda ZAP II vector and packaged into lambda phage	Lambda ZAP II vector	NM	5.5–7.0	55	59.1	GH-43	Wagschal et al., 2009
**11.**	Cow rumen fluid metagenome	GH6284	Lambda ZAP Express and Gigapack II Gold Packaging Extract	Lambda phage library	1/50,000	5.8	45	110.8	GH-5 and GH-26	Palackal et al., 2007
**12.**	Goats ruminal liquid metagenome	Multiple xylanolytic enzymes	CopyControl fosmid library production kit	Fosmid	22/150,000 endo-xylanase 125/150,000 xylosidase	5–7	50	ND	GH-1, GH-5, GH-8, GH-14, and GH-43	Duque et al., 2018
**13.**	Kinema soyabean metagenome	NM	Whole metagenome sequencing	NA	NA	ND	ND	ND	GH39 and GH 43	Kumar et al., 2019
**14.**	Chinese holstein dairy cow rumen	UX66	CopyControl pCClBAC vector and *E. coli*	BAC	18/15,360	6	50	63	GH-43	Zhao et al., 2010
**15.**	Lagoon of dairy farm	Xyn8	Lambda ZAP II Vector/lambda phage	Fosmid	–	6–7	20	45.9	GH-8	Lee et al., 2006

*NM, not mentioned; ND, not determined; NA, not applicable.*

## Strategies to Boost the Retrieval of Metagenomic Xylanolytic Enzymes

Metagenomics demands a rational approach to attain the desired xylanolytic enzymes, and it starts from sample collection to screening to final retrieval of the genes (Figure 1). The following are the few criteria that should be considered for attaining xylan-degrading enzymes from environmental metagenomes.

### Selection of the Xylan Substrates

High-throughput screening strategies enhance the possibility of searching the novel bioactive molecules multifold. For xylanase screening, the traditional Congo red staining method is highly popular; however, it is tedious and labor-intensive, as it demands rigorous replica plating for screening metagenomic libraries. Incorporation of Remazol Brilliant Blue (RBB) cross-linked xylan into the cultivation medium makes the screening efficient, and an appropriately diluted library can be screened for detecting colonies displaying a halo zone of xylan hydrolysis. However, RBB-xylan is quite expensive and can be prepared in the laboratory according to the protocol of Bailey et al. (1992). The fluorescence-based method EnzChek^®^ Ultra Xylanase Assay Kit (Invitrogen, Carlsbad, CA, United States) and Xylazyme tablet (Megazyme, Bray, Ireland) account for the other screening techniques; Azurine-crosslinked xylan (AZCL xylan) is employed as dye conjugated substrates (Selvarajan and Veena, 2017). Several clones have shown a blue halo from fosmid-based metagenomic libraries using AZCL xylan (Ellila et al., 2019; Knapik et al., 2019).

Selection of xylan type is also decisive in obtaining positive clones during functional screening. Birchwood xylan and others are recommended over oat–spelt xylan, Larchwood xylan, and arabinoxylan (Shi H. et al., 2013; Verma et al., 2013a). Birchwood xylan comprises the highest amount of xylose sugar with 89.3% (Kormelink and Voragen, 1993) as compared to beechwood xylan (80.8% xylose; Megazyme) and arabinoxylan (65.8% xylose; Gruppen et al., 1992). Functional screening permits recovery of several full-length metagenomic xylanase-encoding genes by using RBB xylan/AZCL xylan as substrates (Lee et al., 2006; Zhao et al., 2010; Verma and Satynarayana, 2011).

### Fosmid Libraries Are the Preferred Choice

The success rate of functional screening of libraries is significantly higher than the sequence-based approaches in obtaining more positive clones as the latter provides usual partial sequences (Lorenz and Eck, 2005; Berini et al., 2017). Several such metagenomic libraries have been constructed in plasmids (Verma et al., 2013b), cosmids (Mo et al., 2010; Chang et al., 2011; Bao et al., 2012), and fosmids (Jeong et al., 2012; Knapik et al., 2019) for retrieving genes that encode xylan-degrading enzymes (Wang et al., 2015; Kim et al., 2018; Thornbury et al., 2019). Among them, a majority of the metagenomic libraries have been constructed in the fosmid vectors (pCC2FOS^TM^ and pCC1FOS^TM^) with its host strain EPI300-T1R^TM^, provided by Epicentre, for obtaining genes encoding xylan-degrading enzymes (Jeong et al., 2012; Kim et al., 2018; Liu et al., 2019). Fosmids can harbor large stretches of DNA (40–60 kb) that enhance the number of positive hits. Alves et al. (2020) report three potential candidates for xylanolytic enzymes (MgrBr18, MgrBr61, and MgrBr135) out of the 12,960 fosmid clones having an average insert size of 40 Kb. Similarly, 17 novel positive xylanolytic clones were obtained out of 1,15,200 clones from a fosmid-based library (average inert size 30.5 kb). Of the 17 clones, one clone, KG42, revealed the highest xylanase (GH-10 family) activity under acidic conditions (Kim et al., 2018). In another report, a pCT3FK fosmid vector was used to construct 1,50,000 clones, and one positive clone (XylA3) has been identified for xylanase activity (Knapik et al., 2019). Rashamuse et al. (2016) identified several glycosyl hydrolase-positive clones from a fosmid-based metagenomic library of 40,000 clones. The high positive hit rate was also achieved by Liu et al. (2019) on large-scale screening of approximately 50,000 fosmid clones, in which 464 displayed polysaccharide hydrolyzing activity. Of these, 101 clones displayed endo-xylanase activity.

Plasmid-based metagenomic libraries have also emerged successfully in retrieving xylanolytic enzyme-encoding genes (Lee et al., 2006; Wang et al., 2015). One positive xylanolytic clone was attained from the p18GFP vector by screening 36,000 clones constructed from a soil–compost metagenomic library (Verma et al., 2013b). The success rate of plasmid-based metagenomic libraries is low as compared to the fosmid- and cosmid-based libraries; this is, however, one of the cost-effective processes of routine molecular biology laboratories in digging out novel bioactive molecules.

### Mining Genes Using Degenerate Primers

PCR is another popular approach for exploring community DNA cost-effectively for retrieving hidden genes. Several such reports are available in which degenerate as well as specific sets of primers have been successfully used for amplifying novel xylanase-encoding genes from the metagenomes (Liu et al., 2005; Helianti, 2007; Sheng et al., 2015). The degenerate primers often enhance the possibility of fishing out novel candidates over the specific primers (Sheng et al., 2015). In a pioneering report, Sunna and Bergquist (2003) employed GW-PCR to amplify hidden xylanase genes from the hot pool metagenome in New Zealand. A total of 60 xylanolytic genes have been amplified from the gut metagenome of *Holotrichia parallela* larvae using degenerate primers, in which 19 gene products belonged to the GH-10 family, 14 were of GH-11 type, and the remaining genes showed similarity with the sequences of the GH-8 family (Sheng et al., 2015). Similarly, the metagenome of Alpine tundra soil revealed 96 different xylanases of the GH-10 family using a primer based approach, and 31 showed similarity with the members of the GH-11 family (Wang et al., 2010).

Metagenomes pose an issue for the occurrence of a low abundance of the representative genes, especially from extreme environments due to low microbial biomass. To overcome this, rolling circle amplification has been successfully employed to achieve 11 unique putative GH-10 xylanase genes (Yamada et al., 2008). One major drawback associated with PCR-based approaches is retrieving partial genes that chiefly remain uncharacterized.

### Library Sequencing/Whole Metagenome Sequencing

Direct sequencing of the randomly picked clones from a metagenomic library is a straightforward approach to decode the hidden information in the sequences. This approach has also been employed in generating significant information for hundreds of xylanolytic enzyme-encoding genes (Sato et al., 2017; Ariaeenejad et al., 2019). The direct sequencing of a porcupine metagenome uncovered four genes having β-glucosidase, α-L-arabinofuranosidase, β-xylosidase, and endo-1,4-β-xylanase activities (Thornbury et al., 2019). Whole metagenome sequencing is a relatively more powerful technique over library sequencing to construe the hidden insights of the microbial communities as well as their functional characteristics (Sharpton, 2014; Wiseschart et al., 2019). This technique has been adopted to decipher xylan-depolymerizing enzyme-encoding genes from different environmental metagenomes (Wang et al., 2015; Haro-Moreno et al., 2018; Lee et al., 2018; Victorica et al., 2020). Wang et al. (2015) reported four β-xylosidases using shotgun sequencing of compost metagenome. A unique bifunctional cellulase–xylanase was identified by shotgun sequencing of a goat rumen metagenome (Lee et al., 2018). Although a sequence-based approach often emerges with a plethora of unique information, it demands extensive bioinformatics analysis over functional screening.

## Protein Engineering of Metagenomic Endoxylanase

The metagenomic approach has been used to obtain the best-fit enzymes from metagenomes. Several reports focus on genetic manipulations in order to improve the characteristics of metagenomic xylanolytic enzymes (Verma et al., 2019). Unusual metagenomic xylanase (Mxyl) is further improved by site-directed mutagenesis in which four cumulative replacements of surface serine and threonine residues with arginine enhanced the T_1__/__2_ of the muteins by 10 and 5 min at 80 and 90°C, respectively (Verma et al., 2013b). Similar findings have been reported by Turunen et al. (2002), who replaced multiple serine/threonine surface residues of *Trichoderma reesei* xylanase with arginine. The high pKa value of arginine enhances the overall hydrophobicity of the mutated xylanases (Verma et al., 2013a). Such multiple mutations have not been reported for enhancing the thermo-tolerance of endoxylanase II; this also shifted its optimum pH from neutral to the alkaline region. Sriprang et al. (2006) also report the improvement of xylanases using site-directed mutagenesis by multiple substitutions of arginine on protein surfaces. Another metagenome-derived xylanase (Xyn7) was engineered using error-prone PCR, wherein two mutants (XYL7-TC and XYL7-TS) showed a shift in optimum temperature by 10°C with an overall enhancement of 250-fold in their half-lives at 55°C (Qian et al., 2015). The generation of multiple hydrophobic interactions in mutated xylanase, especially between Met50 and other aromatic amino acids, improved the thermostability significantly (Qian et al., 2015). A recently developed *in silico* web tool, TAXyl, assists in predicting the thermal attributes associated with thermophilic and hyperthermophilic xylanases (Shahraki et al., 2019). There are a few reports on the engineering of metagenomic xylanolytic enzymes; a majority of metagenomic xylanases are still awaiting their characterization.

## Sample Sources and the Characteristics of Metagenomic Xylanolytic Enzymes

Soil (Li et al., 2019), compost (Jeong et al., 2012), and gut (Shi et al., 2011) are the three most highly explored habitats for harvesting xylan-degrading enzyme-encoding genes in metagenomics. Compost soil grabbed attention due to the high load of microbial biomass and unique biochemical properties. Jeong et al. (2012) exploited a compost metagenome to retrieve xylanolytic enzyme-encoding genes. A majority of the xylan-hydrolyzing enzymes recovered from the compost displayed optimum activity in acidic/neutral conditions (Matsuzawa et al., 2015; Sun et al., 2015). Kwon et al. (2010) recovered five acidic and thermophilic xylanase-encoding genes from pig manure and mushroom cultivation residues. A novel xylanase-encoding gene (*Mxyl*) from a compost–soil metagenome was found suitable for bleaching of paper pulp samples. This is one of the rare xylanases that exhibits optimum activity at 80°C and alkaline pH of 9.0 with exceptional thermo-stability [T_1__/__2_ (80°C) 120 min)] and alkalistability (pH 9.0), the desired conditions for bio-bleaching of paper pulp samples (Verma et al., 2013b). A majority of the existing xylanases do not exhibit such twin stabilities from culturable microbes (Shi et al., 2014; Yan et al., 2017). A few reports discuss the stabilities of xylanases at higher temperatures and pH, obtained through traditional cultural approaches (Mamo et al., 2006; Verma and Satyanarayana, 2012; Kumar and Satyanarayana, 2014). Several xylanases have been recovered from metagenomes having an optimum temperature of 70°C or beyond (Sunna and Bergquist, 2003; Kanokratana et al., 2014; Sun et al., 2015; Amel et al., 2016; Ellila et al., 2019; Fredriksen et al., 2019; Knapik et al., 2019). However, the majority of them exhibit their optimum pH from acidic to neutral range (5.0–7.0) (Mo et al., 2010; Ellila et al., 2019).

Thermostable and acid-stable xylanases have direct applicability in food and feed industries as well as extraction/clarification of fruit juices (Ngara and Zhang, 2018; Verma et al., 2019). Similarly, several alkaliphilic xylanases have been discovered from direct cloning of environmental DNA (Hu et al., 2008; Thidarat et al., 2012; Ariaeenejad et al., 2019; Liu et al., 2019). Camel rumen metagenome-derived xylanase (PersiXyn1) also shows an optimum pH of 8.0; it, however, exhibits optimum activity at 40°C (Ariaeenejad et al., 2019). Metagenomic xylanases reported by Thidarat et al. (2012) and Liu et al. (2019) share similar properties having optimum temperature in the range of 55–60°C and a pH of 8.0. Interestingly, both of these xylanases have been recovered from termite gut metagenomes.

The gut of insects and herbivores is the well-explored reservoir for a variety of lignocellulose-degrading enzymes (Leth et al., 2018; Table 1). Shi et al. (2011) stress exploring Orthoptera (grasshoppers) and Coleoptera (woodborers) over the Lepidoptera (leaf-consuming insects) to harvest more potent xylanases. It indicates that these insects degrade a more complex form of lignocellulosic materials. Comparative characteristics of the majority of the insect gut xylanases uncovered are acidic and thermophilic xylanases (Brennan et al., 2004; Qian et al., 2015). GH-11 xylanase (xyl7) from termite gut shows enzymatic activity in a broad range of pH (Qian et al., 2015). Similarly, Sheng et al. (2015) recovered numerous clones having xylanase activity from the gut of *H. parallela* larvae, in which a total of 19 GH-10, 14 GH-11, and 27 GH-8 xylanases were identified.

Halophilic/halotolerant xylanases show immense applications in the processing of seafood and clarification of juices and wine (Wang et al., 2016; Alokika et al., 2018). Metagenomics has dug out numerous halotolerant xylanolytic enzymes from different environmental sources (Al-Darkazali et al., 2017; Fredriksen et al., 2019; Ghadikolaei et al., 2019; Li et al., 2019; Alves et al., 2020). Metagenomic xylanase from alkaline-saline soil has shown the ability to retain 80% of its activity at 3M NaCl. On further characterization, this thermostable xylanase reveals that the surface glutamates (E137 and E139) play a key role in its halotolerance and halostability (Li et al., 2019). Chicken cecum metagenome is also shown to be the source of highly halotolerant xylanase that retains 96% activity at 4M NaCl (Al-Darkazali et al., 2017).

Other environmental samples, such as soil, sediment, water, and effluent, from extreme as well as normal habitats have also been explored for xylan-depolymerizing enzymes (Lee et al., 2006; Hu et al., 2008; Wang et al., 2010; Li et al., 2019; Alves et al., 2020). Several cold-active GH-10 and GH-11 xylanase-encoding genes have been retrieved from tundra soil; xylanases displayed cold adaptation (Wang et al., 2010). In a recent report, three potent xylanolytic clones were recovered from mangrove soil metagenomes (Alves et al., 2020). Overall, the majority of the uncovered metagenomic xylanases to date are acidophilic, and their optimum temperature ranges from mesophilic to thermophilic.

## Unusual Metagenomic Xylanolytic Enzymes

The culturable and metagenomic approaches exploit the same environment for mining the genes; therefore, comparing their properties does not look convincing. We cannot ignore that metagenomics also uncovered several hidden genes that we usually lose by routine microbiological culture-dependent methods. Therefore, it is quite interesting to figure out the characteristics of such xylanases and also identify the new additions in terms of their industrial prospects.

A hot pool environmental metagenome uncovered thermostable xylanases having optimum activity at 100°C; the rarest property among the existing xylanases (Sunna and Bergquist, 2003). Though several features of this xylanase are comparable with that of *Thermotoga* spp., its metagenome did not amplify any of its representative sequences (Thermotogales) on 16S rRNA gene amplification. A majority of the xylanases of *Thermotoga* spp. are remarkable in exhibiting optimum xylanase activity at or above 80°C (Simpson et al., 1991; Sunna and Antranikian, 1997; Shi et al., 2014). Compost soil usually emerges with acidophilic xylanases (Kwon et al., 2010; Sae-Lee and Boonmee, 2014; Ellila et al., 2019). Several metagenomic xylanases display very similar properties and harmonize the acidic characteristics of the compost. However, metagenomic xylanase (Mxyl) is an exception for exhibiting notable thermostability at 80°C and alkalistability (pH 9.0–10.0) (Verma et al., 2013b). This is the only report from metagenomics for exhibiting dual stabilities at higher pH and temperature in an endoxylanase. Despite its high molecular weight, it has been categorized into the GH-11 family on the basis of hydrophobic cluster analysis. The endoxylanase of *Geobacillus thermoleovorans* (Sharma et al., 2007; Verma and Satyanarayana, 2012), *Bacillus halodurans* S7 (Mamo et al., 2006), *B. halodurans* TSEV1 (Kumar and Satyanarayana, 2014), *Microcella alkaliphila* JAM-AC0309 (Kuramochi et al., 2016), and *Geobacillus thermodenitrificans* TSAA1 (Anand et al., 2013) display such twin properties; however, their stabilities compromise at higher pH and temperature in the long-run processes and are suitable for industries, especially biobleaching of pulp. Therefore, Mxyl could be a potential candidate for the paper industry. Another unusual metagenomic xylanase (xyn8) from an environmental DNA library shows a distinct optimum temperature of 20°C, which is quite rare from the culture-dependent approach (Zhao et al., 2010).

A majority of metagenomic psychrophilic xylanases share similar properties with the cold-active xylanases of bacteria/fungi (Lee et al., 2006; Guo et al., 2009; Wang et al., 2010). More efforts are, therefore, needed that suit extreme cold environments for retrieving psychrophilic/psychrotolerant xylanolytic enzymes. Furthermore, a unique bifunctional xylanase/endoglucanase from a yak rumen metagenome has been revealed to exhibit a synergistic effect with β-1,4-xylosidase and β-1,4-glucosidase; these are considered ideal candidates for the bioethanol industry (Chang et al., 2011). The bifunctional xylanases also enhance feed nutrient digestibility multifold over the mono-functional xylanases for use in enzyme cocktails (Khandeparker and Numan, 2008).

The discovery of unique halophilic/halotolerant xylanolytic enzymes further enhances the significance of metagenomics. Camel rumen metagenome revealed a rare halophilic xylanase that gets stimulated by 132% in the presence of 5M NaCl, which is the highest reported among the various other existing xylanases from culturable approaches to date (Ghadikolaei et al., 2019). In a recent investigation, novel xylanase (AMOR_GH10A) was identified from the Arctic Mid-Ocean Ridge vent system that exhibited dual binding affinity on xylan as well as glycans. On sequencing analysis, its carbohydrate-binding domain (CBD) does not share any similarity with any of the existing sequences available in the databases (Fredriksen et al., 2019). This paved the way to search for new candidates of the CBM85 family that have promising substrate affinities. A unique feature has been reported for the first time from an exceptional metagenomic xylanase (UX66) that comprises two CBDs and two catalytic domains (Zhao et al., 2010). Another unique three catalytic domain multi-enzyme (a CE1 ferulic acid esterase, a GH62 α-L-arabinofuranosidase, and a GH10 β-D-1,4-xylanase) has been recovered from the metagenome of wastewater treatment sludge (Holck et al., 2019). Such attributes enhance the applicability of xylanase manifold over the xylanases having one single catalytic domain. Undoubtedly, metagenomics has emerged as an exceptional tool for adding several novel and unique xylan degrading biocatalysts that find potential in different industries.

## Addition of Novel Metagenomic Xylanase-Encoding Genes Into the CAZy Database

**FIGURE 2:**
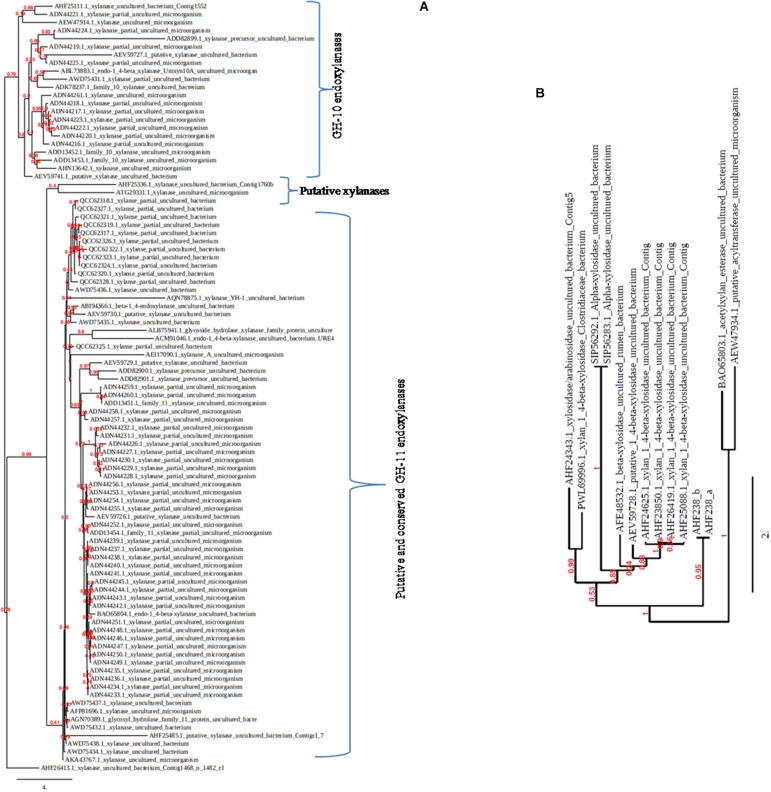
The phylogenetic trees showing the diversity among metagenomic xylanolytic enzymes. The trees (**A:** GH-10 and GH-11 xylanases; **B:** xylanolytic enzymes other than GH-10 and GH-11 xylanases) were constructed by including the protein sequences (complete/partial) available in the NCBI database using the keyword xylanase/xylosidase/arabinofuranosidase/acetyltransferase from uncultured bacteria/microorganisms.

Sequencing analysis of the retrieved metagenomic xylanase proteins uncovers a plethora of genes that were inaccessible earlier. The CAZy database comprises several representatives of metagenomic xylanolytic enzymes that are categorized into GH-10 and GH-11 families. Approximately 470 endo-acting xylanases are present in the GH-10 and GH-11 families of metagenomic origin; most of them are uncharacterized partial fragments of less than 100 amino acids. Similarly, β-xylosidases also account for 28 unclassified candidates. The blastp analysis of these sequences reveals that metagenome-originated xylanases share low/high percentage identity from their homologue sequences available in the databases (Mo et al., 2010; Bastien et al., 2013; Rashamuse et al., 2016; Ellila et al., 2019). The phylogenetic tree further depicts clustering of the sequences (full length/partial) into respective GH families (Figure 2); nine partially redundant endo-active xylanases and five arabinosidase/xylosidase metagenomic fragments with very high identity, in which most of the sequences display similarity with the homologue sequences of Bacteroidetes; an overrepresented group of termite gut (Bastien et al., 2013).

A gene derived from a Hu sheep rumen metagenome shares 97% identity with a contig1552 of an uncultured bacterium; its xylanase, however, shows very little similarity with the homologous proteins (Wang et al., 2019). Metagenomic xylanase Xyn10CD18 shares a maximum identity of 83% with thermostable xylanase of *Bacillus* sp. N16-5 (Sun et al., 2015). Similarly, the xylanase gene retrieved from a Pawan hot spring metagenome also shares an identity of 95% with its protein homologue (accession number: CAA84276) (Helianti, 2007). It may be due to the PCR approach to fetch the desired genes, when the specific and degenerate sets of primers are designed from the available gene sequences (Verma et al., 2019). In addition, numerous metagenomic xylanolytic enzymes have also been identified for showing low to very low percentage identity with their homologues (Ellila et al., 2019). Metagenomic xylanase (Xyn38) shares only 50.3% identity with the GH-10 xylanase of *Acidothermus cellulolyticus* 11B (Ellila et al., 2019). AMOR_GH10A shares a maximum of 42% identity with a hypothetical protein from a *Verrucomicrobia* (Fredriksen et al., 2019). However, its GH-10 domain shows an even lesser identity of 24–28% with the homologous xylanases. Similarly, KG42 (Kim et al., 2018), XYL6806 (Brennan et al., 2004), and xyn10N18 (Gong et al., 2013) xylanases also share a very low identity of merely 40% with the homologue sequences. The discovery of bifunctional cellulase-xylanase also shows a low identity of 41% with Cel5 homologues (Lee et al., 2018).

**FIGURE 3:**
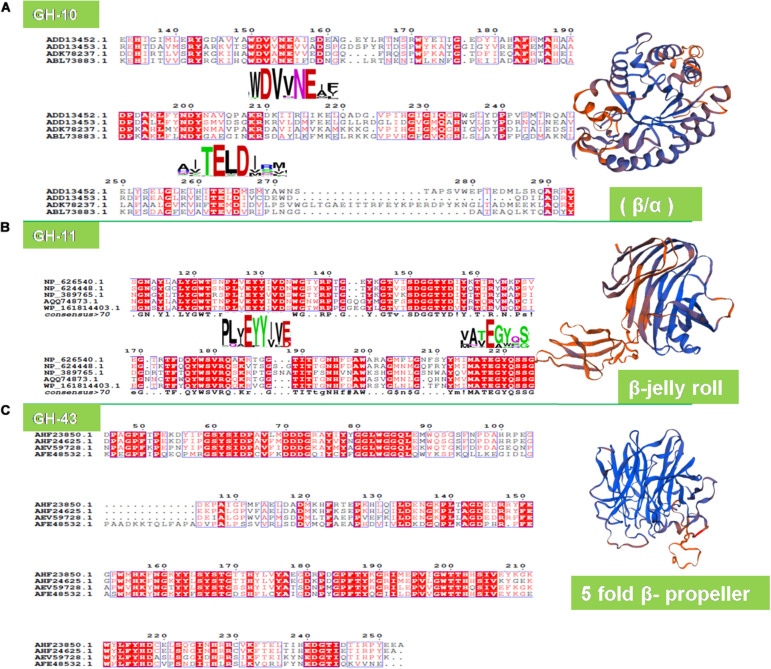
Structure analysis of metagenomic xylanolytic enzymes. The ESPRipt analysis showing the conserved motifs for **(A)** GH-10 (WDVVNEI/A and TEL/MDM/I), **(B)** GH-11 (PLY/AEYYI/VE/D and ATEGYQ) endoxylanases, and **(C)** GH-43 (DDDG and LFYI). The GH-10 and GH-11 xylanases exhibited conserved motifs having highly conserved catalytic glutamate (E) and aspartate (D) residues. The signature structure of metagenomic GH-10 (Accession number: ADK78237.1), GH-11 (accession number: AQQ74873.1), and GH-43 (accession number: AFE48532.1) enzymes.

On the contrary, the metagenomic xylanase (XynH) shares a higher identity of 56% and a similarity of 71% with the GH-10 family xylanase of *Cellvibrio mixtus* (accession number AF049493; Hu et al., 2008). Similarly, umxyn10A shares 58% identity and 73% similarity with xylanase from a bacterium *Thermobifida fusca* (Mo et al., 2010). Five novel xylanase genes having a 35–40% sequence identity have been retrieved from the PCR-based approach using a horse vermiform appendix metagenome, which is quite surprising (Yamada et al., 2008). Multiple sequence alignments, however, have detected the crucial catalytic residues (glutamate and aspartate) in the signature sequences of xylanolytic enzymes of metagenomic origin (Figure 3).

## Metagenomic β-Xylosidases and Their Characteristics

Among xylanolytic enzymes, β-xylosidases (Exo-1, 4-β-D xylosidase E.C. 3.2.1.37) are the second most highly important xylan-depolymerizing enzymes that assist in complete degradation of xylans. These exotype glycosidases cleave the glycosidic linkages of short xylooligosaccharides (XOs) and liberate the pentaose sugar (xylose) as an end product (Shallom and Shoham, 2003; Jain et al., 2015). Pharmaceutical and food industries demand thermostable and halophilic β-xylosidases (Jain et al., 2014). Existing β-xylosidases are associated with several limitations, such as poor efficiency, low thermostability, salt sensitivity, and by-product inhibition (Bao et al., 2012; Anand et al., 2013). Therefore, efforts have been made to discover novel candidates of β-xylosidases using metagenomic approaches, with which little success has been achieved (Wagschal et al., 2009; Jordan et al., 2016; Cheng et al., 2017; Sato et al., 2017; Liu et al., 2018; Rohman et al., 2019; Table 2). Of the available metagenomic β-xylosidases, fewer than 20 have been extensively characterized to date. The majority of such β-xylosidases have been reported from the ruminal fluid of yak (Zhou et al., 2011, 2012; Bao et al., 2012), cow (Jordan et al., 2016), and buffalo (Singh et al., 2014) due to the abundance of hemicellulose-degrading microbes in their guts. Other promising environmental sources have also been explored, such as soil (Matsuzawa et al., 2016) and compost (Sae-Lee and Boonmee, 2014; Matsuzawa et al., 2015; Wang et al., 2015), for harnessing hidden β-xylosidase genes. On characterization, most of the β-xylosidases show optimum pH from an acidic to neutral range (Wagschal et al., 2009; Jordan et al., 2016; Liu et al., 2018). Three highly acidic β-xylosidases (AR19M-311-2, AR19M-311-11, and AR19M311-21) derived from a hot spring metagenome show optimum pH of 5.0 (Sato et al., 2017). The yak rumen metagenome revealed many acidic β-xylosidases, and one of the β-xylosidases (RuBG3A) exhibited activity at pH 4.5 on pNPG. There is only one report of metagenomic β-xylosidase (xylM1989) that shows its optimum activity at an alkaline pH of 8.0 (Maruthamuthu et al., 2017).

Thermostable β-xylosidases are in high demand in industries to overcome microbial contamination and reduce the viscosity of the reaction mixture, which improves the overall reaction efficiency manifold (Haki and Rakshit, 2003). The β-xylosidases (AR19M-311-2, AR19M-311-11, and AR19M311-21) reported by Sato et al. (2017) are highly thermostable having optimum activity at 90°C with fair stability at 70°C for 1 h. It corroborates the characteristics of thermostable β-xylosidases of *Thermoanaerobacter ethanolicus, Thermotoga maritima*, and *Thermotoga thermarum* having the temperature optima at and above 90°C (Shao and Wiegel, 1992; Shi W. et al., 2013). In another report, four thermostable β-xylosidases have been reported from a compost–soil metagenome that show optimum activity at 60–75°C (Wang et al., 2015). In addition, these β-xylosidases are found to retain 80% of residual activity at 50°C after 2 h of incubation. A novel and first metagenomic α-xylosidase (MeXyl31) of the GH-31 family has been reported from a soil metagenome that shows an optimum temperature of 45°C (Matsuzawa et al., 2016).

Several β-xylosidases and metagenomic β-xylosidases have also been identified for their bifunctional enzymatic activities (DeCastro et al., 2016; Rohman et al., 2019). Such β-xylosidases find applicability in bioethanol production along with the endo-xylanases for efficient release of sugars from the hemicellulose component of agro-residues (Sae-Lee and Boonmee, 2014; Wang et al., 2015). The bifunctional β-glucosidase/xylosidase activity has been observed in Rubg3A, Rubg3B, and RubGX1 (Zhou et al., 2011; Bao et al., 2012). Many β-xylosidases derived from the functional screening of the metagenomic library also detected arabinofuranosidase activity (Wagschal et al., 2009; Wang et al., 2015; Jordan et al., 2016). On characterization, β-xylosidases/arabinofuranosidase (RUM630-BX) enhanced activity 84-fold due to the stimulation by divalent metal ions (Ca^2+^, Co^2+^, Fe^2+^, Mg^2+^, Mn^2+^, and Ni^2+^) (Jordan et al., 2016). Jordan et al. (2018) revealed the role of aspartate and histidine residues at the active site that chelates Ca^2+^. Therefore, on supplementation of the divalent ions, the overall β-xylosidase activity was restored. The termite gut metagenome revealed a multimeric β-xylosidase having a catalytic activity of β-glucosidase or β-arabinosidases among four of the positive clones (Liu et al., 2018). A majority of the reported metagenomic β-xylosidases can be considered as novel due to their unique features along with very low sequence identity/similarity with their homologues.

## Metagenomic Arabinofuranosidase and Acetyl Xylan Esterase

α-L-arabinofuranosidase (EC 3.2.1.55) represents another important class of hemicellulose-degrading enzymes that release arabinofuranosyl side moieties from a heteroxylan by cleaving α-1,2 and α-1,3 glycosidic bonds (Bouraoui et al., 2013; Kumar et al., 2013b). In addition, non-acetylated heteroxylans have direct access for their backbone-hydrolyzing enzymes, such as endoxylanases and β-xylosidases (Rennie and Scheller, 2014). More often, α-L-arabinofuranosidase acts synergistically along with the cocktail of different xylanolytic enzymes. It finds applications in biomass conversion, food and feed industries, and environmental waste management (Wilkens et al., 2017; Verma et al., 2019). Metagenomic approaches have brought out very few α-L-arabinofuranosidases from different environmental sources (Fortune et al., 2019). Recently, three α-L-arabinofuranosidases (AFaseH4, AFaseE3, and AFaseD3) have been reported from the high-temperature compost metagenome (Matsuzawa et al., 2015; Fortune et al., 2019). On characterization, all three display optimum activity at pH in the acidic range of pH 4.0–5.0 and at 40°C. However, sequence analysis reveals that AFaseH4- and AFaseE3-encoding genes share 100 and 99% identity with the existing sequences, respectively, while the ORF-encoding AFaseD3 shares 77% identity with the AFAse of a *Paenibacillus taihuensis* ORF (Kolinko et al., 2018). Many other metagenomic arabinofuranosidases have shown activity with β-xylosidases as bifunctional enzymes (Wagschal et al., 2009; Wang et al., 2015; Jordan et al., 2016).

The acetyl xylan esterases (AXEs) (EC 3.1.1.6) cleave the acetyl group from acetyl-xylose moieties and contribute to the complete degradation of the complex xylans. Recently, a few studies have uncovered a handful of AXEs using metagenomic approaches (Adesioye et al., 2018; Brito et al., 2018; Table 2). A metagenomic AXE (Axe1NaM1) was identified by using a hot desert hypolith metagenomic DNA sequence data set (Adesioye et al., 2018). On characterization, it was detected as a mesophilic (Topt. 30°C) and alkaliphilic AXE (pHopt. 8.5). Interestingly, a point mutation (N65S) improved its thermostability as well as catalytic efficiency; the crystal structure of this enzyme has also been solved. In another report, the gut microbiome of a shipworm also identified several AXEs that shared 50–75% similarity with the available carbohydrate esterases (Brito et al., 2018). Zhu et al. (2016) studied the association and relative abundance of *Firmicutes* along with lignocellulose-degrading enzymes.

## Utility of Metagenomic Xylanolytic Enzymes

Xylanolytic extremozymes find extensive applications in various industries due to their high performance under extreme conditions (Kumar et al., 2016; Figure 1). Unfortunately, a majority of the metagenomic xylanolytic enzymes are limited to biophysical characteristics only (Gong et al., 2013; Kanokratana et al., 2014; Matsuzawa et al., 2015; Ghadikolaei et al., 2019). A handful of metagenome-derived xylan-degrading enzymes have been explored for their applicability in various industrial processes (Jeong et al., 2012; Wang et al., 2015; Berini et al., 2017). The metagenomic xylanase (Mxyl) was successfully employed for bleaching paper pulp samples in the paper industry, and an approximately 24% reduction in chlorine consumption was achieved (Verma et al., 2013a). Similarly, Ariaeenejad et al. (2019) also report the applicability of camel rumen metagenome–derived xylanase (PersiXyn1) in biobleaching of carton paper pulp. The recombinant enzyme (Mxyl) also finds application in saccharification of agro-residues (wheat bran, corn cob, sugarcane bagasse) for generating XOs of low DP of 2–4 xylose units (Verma et al., 2013b). Such XOs exhibit a prebiotic effect by promoting the growth of probiotic gut microbiota. The xylanase CoXyl43 shows synergistic action with cellulase of *T. reesei* in enhancing its saccharification efficiency on rice straw (Matsuzawa et al., 2015). Sun et al. (2015) achieved an overall 80% XOs (DP 2–4) from corn cob hemicellulose bioconversion by using metagenome-derived Xyn10CD.

The cocktail of β-xylosidases (RuBG3A/RuBG3B) along with endoxylanases facilitate hemicellulose saccharification efficiently (Bao et al., 2012). Another metagenome-derived cocktail of four thermostable xylanolytic enzymes (β-xylosidase and β-xylosidases/α-arabinofuranosidase) was able to hydrolyze approximately 55% of the steam-exploded corn cobs at 50°C in 48 h (Wang et al., 2015). Duque et al. (2018) claim that the metagenomic β-xylosidases are tenfold more efficient than the commercial fungal cocktails of the xylanolytic enzymes. However, Jeong et al. (2012) suggest supplementation of metagenomic xylanases (Xyn10J) with the commercial enzymes to improve their saccharification efficiency. In another report, a compost-derived xylanase (XYL40) exhibited higher hydrolysis than the xylanase of TrXyn11A (*T. reesei* xylanase). Sae-Lee and Boonmee (2014) propose that thermophilic Biof1_09 protein (with dual cellulase and xylanase) can be employed in stone washing and biopolishing industries that require thermostable and acidic extremozymes. Thermophilic xylanase and endoglucanase derived from sugarcane bagasse metagenome enhances the efficiency of a commercial cellulase Celluclast^®^ (Novozymes, Bagsvaerd, Denmark) (Kanokratana et al., 2014). Kim et al. (2018) report the prebiotic effect of xylan hydrolysates on the growth of gut bacterial strains (*Bifidobacterium longum* and *Bifidobacterium lactis*). Interestingly, this metagenome-derived thermostable xylanase (KG42) significantly enhances the overall growth of these strains by 95–97%.

A few patents have also described various attributes of metagenome-derived xylan-degrading enzymes. Thermophilic xylanase from a hot spring metagenome has been patented (Patent no: EP2990482 A1) for its application in biofuel production from lignocellulosic biomass (Berini et al., 2017). Two Indian patents (Patent Appl. Nos. 201811041913 and 201711020622) were also filed in 2018 on process devolvement for XO production using metagenome-derived novel xylanases. Radomski et al. (1998) patented a method for retrieving the xylanase gene from soil metagenome (United States Patent US 5849491).

## Conclusion and Future Perspectives

It has taken more than 15 years to report the first xylanase by Sunna and Bergquist (2003) using a culture-independent approach. Since then, the CAZy database is being enriched by several of the homologue sequences, where the count of GH-10 and GH-11 xylanases dominates the other xylan-degrading proteins. The supremacy of GH-10 xylanases clearly indicates their broad substrate specificity as compared to the GH-11 (true xylanases) members that are quite selective in their substrate range. A majority of the uncovered xylanolytic enzymes are of thermo-acidophilic type; this might be due to their selection from such environments. Better candidates can be recovered by employing a rational approach that includes high-throughput screening of fosmid-derived metagenomic libraries generated from extreme environmental metagenomes on suitable dye-conjugated xylan substrates. In addition, enrichment of cultures under desired physical conditions along with suitable substrates also enhances the success rate multifold to achieve xylanases with the requisite properties. Complementing shotgun sequencing and a PCR-based approach enhances the possibility of retrieving novel xylanolytic extremozymes from the environmental metagenomes.

Undoubtedly, metagenomics has uncovered several novel xylanolytic proteins having unusual properties that were earlier inaccessible. However, it cannot be denied that the recovery of metagenomic xylanolytic enzymes is comparatively higher, and their characterization and industrial applications must be stressed. A handful of reports discuss the feasibility of the retrieved enzymes at the industrial level. We have achieved very little success in utilizing these enzymes. Megazyme^2^ commercialized an endo-1,4-β-xylanase (from a rumen microorganism) that finds applications in food/feed as well as in paper pulp industries. Similarly, Luminase, a commercial product of BASF^3^ is also used for biobleaching in paper industries (Berini et al., 2017). We, therefore, emphasize more on characterizing the various available metagenomic xylanases for their applicability and screening for the new candidates.

## Author Contributions

Both authors wrote and finalized the manuscript.

## Conflict of Interest

The authors declare that the research was conducted in the absence of any commercial or financial relationships that could be construed as a potential conflict of interest.

## Footnotes

1 www.cazydatabse.com

2 www.megazyme.com

3 www.basf.com
